# Perceived Discrimination and Substance Use among Caribbean Black Youth; Gender Differences

**DOI:** 10.3390/brainsci8070131

**Published:** 2018-07-09

**Authors:** Shervin Assari, Ritesh Mistry, Cleopatra Howard Caldwell

**Affiliations:** 1 Center for Research on Ethnicity, Culture and Health, School of Public Health, University of Michigan, Ann Arbor, MI 48109, USA; cleoc@umich.edu; 2 Department of Psychiatry, University of Michigan, Ann Arbor, MI 48109, USA; 3 Department of Health Behavior and Health Education, School of Public Health, University of Michigan, Ann Arbor, MI 48109, USA; riteshm@umich.edu

**Keywords:** ethnic groups, Blacks, Caribbean Black, racial discrimination, smoking, substance use, gender

## Abstract

Although perceived discrimination in Black youth is a risk factor for a wide range of negative mental health outcomes, recent research has suggested some gender differences in these associations. Gender differences in vulnerability to perceived discrimination among Caribbean Black youth is, however, still unknown. The current cross-sectional study investigated gender variations in the association between perceived discrimination and substance use (SU) in a national sample of Caribbean Black youth. Data came from the National Survey of American Life-Adolescents (NSAL-A), 2003–2004. This analysis included 360 Caribbean Black youth (165 males and 195 females) who were between 13 and 17 years old. Sociodemographic factors, perceived discrimination, and SU were measured. Logistic regressions were used for data analysis. Among Caribbean Black youth, a positive association was found between perceived discrimination and SU (odds ratio (OR) = 1.15 (95% confidence interval (CI) = 1.02–1.29)). A significant interaction was found between gender and perceived discrimination on smoking (OR = 1.23 (95% CI = 1.07–1.41)) suggesting that the association between perceived discrimination and smoking is larger for male than female Caribbean Black youth. The interaction between gender and perceived discrimination on SU was not statistically significant (OR = 1.32 (95% CI = 0.94–1.86)). While perceived discrimination increases SU in Caribbean Black youth, this effect is stronger for males than females, especially for smoking. While discrimination should be reduced at all levels and for all populations, clinicians may specifically address discrimination for SU prevention and treatment among male Caribbean Black youth.

## 1. Introduction

Discrimination is a risk factor for poor mental health [1,2,3,4,5,6]. At least some racial/ethnic health disparities are due to racial discrimination [5,7,8,9,10,11,12,13,14]. Black adults [3,15] and Black youth [16,17] perceive high levels of discrimination on a daily basis. Perceived discrimination is a risk factor for a wide range of negative physical, mental, and behavioral health outcomes including but not limited to depression [18], negative affect [15], anxiety [18], suicide [19], smoking [20], and substance use (SU) [21,22].

### 1.1. Gender Differences

Research suggests that among racial/ethnic minorities, males perceive more discrimination than females. Among Blacks for example, males report higher levels of perceived discrimination compared to females [16,23,24,25,26,27], a phenomenon that can be explained by the subordinate male hypothesis [28]. Based on this hypothesis, the discrimination experienced by the men of subordinate groups is greater than the discrimination experienced by the women of the same subordinate groups [28]. Based on this hypothesis, gender interacts with race/ethnicity in shaping exposure to discrimination. This hypothesis is supported by a recent study that documented higher levels of implicit bias against Blacks in White men compared to White women [29]. American society has stereotyped Black males as aggressive and anti-intellectual [25,27,30,31]. Black males have also been stereotyped as endangered, superhuman, subhuman, lazy, lost, loveless, incorrigible, aggressive, angry, self-destructive, and hyperactive [32,33]. Several social factors—such as police brutality, mass incarceration, and neighborhood crime—also disproportionately impact Black males more than any other sociodemographic group [34,35]. Black males are also more likely to be discriminated against by teachers and are at a higher risk of school drop-out, which contributes to a school to prison pipeline for Black males [25,36,37,38,39,40]. Finally, Black males receive more racial socialization messages from parents compared to Black females [41,42], which in turn increases their awareness of racism as well as vigilance toward discrimination.

Gender also alters some of the health correlates of perceived discrimination [18,43,44,45]. In an 18-year longitudinal study, an incremental increase in discrimination during adolescence was predictive of a subsequent increase in symptoms of anxiety and depression for male but not female Black adolescents [18]. In another study, perceived discrimination was a risk factor for psychological distress for male but not female Arab Americans [43]. Brondolo also showed that males may be more prone to discrimination than females [46]. These studies collectively suggest that discrimination may have a more salient role as a risk factor of SU and poor mental health for males than females [43,45], a phenomenon that may be due to gender norms such as hegemonic masculinity beliefs (traditional gender norms) [47].

Most research on the link between perceived discrimination and mental/behavioral health has focused on the overall effects of discriminatory experiences and outcomes rather than factors that may mitigate such associations (i.e., vulnerability factors) [48]. In addition, more is known about the effects of perceived discrimination on emotional problems such as depression and anxiety [11,18,49,50] than behavioral outcomes such as SU [46]. Moreover, research has frequently enrolled local samples that generate non-generalizable results [46]. Finally, we know less about these associations in adolescents than adults.

### 1.2. Mechanisms

Health effects of discrimination have been attributed to a number of mechanisms including: (1) negative emotions such as distress, perceived stress, and worries; (2) heightened chronic stress response; (3) hyper-vigilance; (4) social isolation; and (5) health risk behaviors such as SU [11,23,51,52]. Gibbons [53,54,55] and Brondolo [46] have addressed the theoretical underpinnings of the harmful effects of discrimination among Black youth and adults. In a long-term cohort study of Black youth, Gibbons showed that anger [53], externalizing reactions [55], and reduced self-control [54] play some role in explaining the association between perceived racial discrimination and SU. In separate studies by Gibbons, affect, anger, perceived control, and externalizing reactions were shown as mediating factors between experiences of discrimination and SU among Black youth [53,54,55]. Brondolo categorized experiences of discrimination into five types: exclusion/rejection, stigmatization, threat/aggression, work/school, and lifetime discrimination. The relative importance of these types of discriminations may be different for males and females [56]. Sanders-Phillips documented the mediating role of depression and distress in the association between discrimination and SU [57].

### 1.3. Caribbean Blacks

The Black community is ethnically diverse, and each ethnic group of Blacks has unique and distinct history, culture, and life experiences. Despite such heterogeneity, very little is known about the variability in experiences and consequences of discrimination among various subgroups of Blacks. Caribbean Americans account for about 9% of the immigrant population in the U.S. and 50% of them self-identify as Black. Caribbean Blacks are the largest Black immigrant group and the main source of growth of the Black population in the U.S. Though Caribbean Blacks also historically faced slavery, colonies in the Caribbean banned slavery decades earlier than the U.S. Afro-Caribbean is the most common ethnic group in many Caribbean countries. The historical racism experienced by Blacks in The Caribbean and Caribbean Blacks in the U.S. is qualitatively different from the experiences of African Americans. Therefore, it is important to examine Caribbean Blacks separately in order to better understand the role of discrimination in diverse Black communities [58].

Caribbean Black youth differ from their African American counterparts. Cultural and SES differences and the issues of immigration discrimination and gender role norms may operate differently within Caribbean Black and African American families. For example, psychosocial determinants of discrimination, physical health, and mental health for Caribbean Black youth differs from those of African American youth [59,60,61,62]. Gender differences in the epidemiology of psychiatric disorders are reversed for Caribbean Black and African American youth in the NSAL. While in African American youth, depression and anxiety were more common for females than males, depression and anxiety were more common for male than female Caribbean Black youth [63]. Finally, most Caribbean Blacks in the U.S. are more recent generations of immigrants [63]. Due to these differences, we examine Caribbean youth because we expect that social and behavioral correlates of discrimination would also be unique for them. Still, very little is known about discriminatory experiences of Caribbean Black youth in the U.S.

Using a national sample, the current study investigated the association between perceived discrimination and SU among Caribbean Black youth by gender. Given males’ higher exposure [64] and vulnerability to the effects of discrimination on behaviors and distress [43,46,65] in other race/ethnic minority groups, we hypothesized that the association between perceived discrimination and SU would be stronger for male than female Caribbean Black youth.

## 2. Methods

### 2.1. Design and Setting

This study used cross-sectional data from the National Survey of American Life-Adolescent Supplement (NSAL-A), 2001–2003 [17,66]. NSAL, a part of the Collaborative Psychiatric Epidemiology Surveys (CPES), 2001–2003 [67], is one of very few available mental health surveys of Black American youth, with a nationally representative sample. Funded by the National Institute of Mental Health (NIMH), the NSAL was conducted by University of Michigan (UM), Ann Arbor [68].

### 2.2. Ethics

The University of Michigan (UM) Institute Review Board (IRB) approved the study protocol (IRB number B03-00004038-R1). Informed consent was received from youths’ legal guardians. All youth provided assent. Participants were financially compensated ($50). All procedures in the study were in accordance with the ethical standards of the institutional and/or national research committee.

### 2.3. Participants

The current study included a total number of 360 Caribbean Black youth. All participants were ages 13 to 17 years old. All participants resided in the U.S. territories at the time of participation in the study. Additional details regarding sampling are published elsewhere [67,68].

### 2.4. Samples and Sampling

The NSAL-A sample was drawn from the NSAL-Adults. The NSAL-Adults used a national probability sample of Black adults in the United States. In the first step, the NSAL households were screened for eligible adolescents living in the household. In the next step, adolescents were randomly selected for participation in the NSAL-Adolescent study. In the event that more than one eligible adolescent lived in the household, two adolescents were selected to the study based on the gender of the first eligible youth. The second youth selected was of the opposite gender of the first youth. This issue resulted in non-independence of the NSAL-A sample. To adjust for lack of independence, as well as selection probabilities and non-response at the household and individual levels, the NSAL-A data were weighted. At the last step, the weighted data were post-stratified to represent national figures based on age, gender, and ethnicity [17].

### 2.5. Interview and Data Collection

Data were collected using interviews that were performed in English and took 100 min on average. The response rate of the NSAL-A was 83.5% for Caribbean Black youth. About 82% of all interviews were in-person interviews, performed in the adolescents’ homes. The remaining 18% of the interviews were entirely or partially conducted via phone. Computer Assisted Personal Interview (CAPI) was used for all the in-person interviews [69].

### 2.6. Study Measures

Demographic Factors: The current study included demographic factors including age and gender. Age was a continuous measure. Gender was a dichotomous measure (male = 1 and female = 0).

Socioeconomic Status (SES): SES was measured as poverty index, defined as the household income divided by the poverty threshold for similar households, based on the 2001 U.S. Census thresholds [70]. Higher poverty index reflected higher SES.

Race and Ethnicity: The NSAL-A defined youth race and ethnicity based on the self-identified race and ethnicity of the parents and guardians in the household where the youth lived. Caribbean Black was defined as Black individuals who had some ancestral tie to any Caribbean countries. The interviewer provided a list of Caribbean countries to parents. Countries included: Antigua and Barbuda, Barbados, Bahamas, Cuba, Dominican Republic, Dominica, Grenada, Haiti, Jamaica, Saint Vincent and the Grenadines, Trinidad and Tobago, Saint Lucia, and Saint Kitts and Nevis.

Perceived Everyday Discrimination: NSAL-A has used a modified version of the Everyday Discrimination Scale (EDS) to assess perceived discrimination. Sample items included: “being followed around in stores”, “receiving poorer service than other people at restaurants”, and “being called names or insulted”. This measure assesses chronic, routine, and less overt discriminatory experiences that have occurred over the past year [71]. Although the original EDS has 10 items, the NSAL-A EDS had 13 items. The additional three items covered perceived discrimination by teacher. EDS is normed and validated in adults as well as adolescents. The measure has very good psychometric properties in youth [17,71]. The items used a Likert responses scale ranging from 1 (never) to 6 (almost every day). We calculated a sum score that reflected frequency of exposure to discrimination over the past year. A higher score was indicative of more discrimination (α = 0.86).

Substance Use (SU): To measure lifetime illicit SU, all the participants were asked “Have you ever used …?” The substances that were assessed included marijuana/hashish, cocaine (in any form, including powder, crack, free base, coca leaves, or paste), prescription drugs (tranquilizers, stimulants, pain killers) without the prescription of a health professional, or for reasons other than a health problem, and/or other drugs (heroin, opium, glue, Lysergic acid diethylamide (LSD), peyote, or any other drug). We calculated an aggregate based on all the above items. SU was treated as dichotomous variables, which reflects a yes response to any SU.

Smoking: Lifetime smoking was measured using the following single item: “Have you ever smoked a cigarette, cigar, or pipe, even a single puff?” Participants responded yes or no to the question. Single item measures have been commonly used to study smoking and SU in epidemiological surveys [72,73,74].

### 2.7. Statistical Analysis

We used Stata 13.0 (Stata Corp., College Station, TX, USA) to accommodate for the NSAL-A complex design. We reported weighted proportions (relative frequencies) and means for descriptive purposes. We also reported Pearson correlation r values in the pooled sample and by gender.

We used the Taylor expansion approximation technique to re-calculate the complex design-based estimates of variance. Standard errors reflect the weights due to the complex sampling design. All percentages reported in this study are weighted.

We used sub-population survey logistic regressions for our multivariable analyses. In our logistic regression models, perceived discrimination was the independent variable, either smoking or SU were the dependent variables, and age, gender, and poverty index were covariates. First, the association between perceived discrimination and SU/smoking was estimated in all Caribbean Black youth. In the next step, we added the interaction term between discrimination and gender. Finally, we ran logistic regression models specific to gender.

Adjusted odds ratios (OR) and their 95% confidence interval (CI) were reported for our multivariable analyses. *p* values smaller than 0.05 was regarded as statistically significant.

## 3. Results

### 3.1. Descriptive Statistics

This study included 360 Caribbean Black youth who were either male (*n* = 165) or female (*n =* 195). Table 1 summarizes descriptive statistics in the sample overall and by gender. Perceived discrimination was found to be higher in males than females, while SU and smoking were higher in females than males.

### 3.2. Bivariate Correlations

Table 2 reports the correlation matrix between sociodemographic factors, perceived discrimination, smoking, and SU in the pooled sample and by gender. Smoking and SU were positively correlated in males and females. In both genders, age showed a moderate positively correlation with SU.

Table 3 summarizes the results of two logistic regression models in the overall sample of Caribbean Black youth, with SU as the outcome, perceived discrimination as the predictor, and age, gender, and poverty index as covariates. Model 1 only included the main effects without any interaction term. Model 2, however, also included an interaction term between gender and perceived discrimination. Perceived discrimination was positively associated with SU in the pooled sample. The interaction between gender and perceived discrimination on SU was, however, not statistically significant (OR = 1.32 (95% CI = 0.94–1.86)) (Table 3).

Table 3 also summarizes the results of logistic regression models with perceived discrimination as the independent variable and SU as the dependent variable by gender. Higher level of perceived discrimination was associated with higher odds of SU in males (OR = 1.32 (95% CI = 1.05–1.67)) but not females (OR = 1.02 (95% CI = 0.91–1.15)).

### 3.3. Logistic Regressions for Smoking

Table 4 summarizes the results of two logistic regression models in the pooled sample of Caribbean Black youth, with smoking as the outcome, perceived discrimination as the predictor, and age, gender, and the poverty index as covariates. Model 1 only included main effects. In Model 2, we included an interaction term between gender and perceived discrimination. In the pooled sample of Caribbean Black youth, perceived discrimination was not associated with higher odds of smoking (OR = 1.07 (95% CI = 0.77–1.49)). A significant interaction was found between gender and perceived discrimination on smoking (OR = 1.23 (95% CI = 1.07–1.41)) suggesting that the association between perceived discrimination and smoking is larger for male than female Caribbean Black youth.

Table 4 also summarizes the results of two logistic regression models with perceived discrimination as the independent variable and smoking as the dependent variable, in each gender. Higher level of perceived discrimination was not significantly associated with odds of smoking in males or females.

## 4. Discussion

Using an observational design, we found some evidence suggesting that gender may alter the magnitude of the effects of perceived discrimination on smoking and SU among Caribbean Black youth. There was a significant interaction between gender and perceived discrimination on smoking indicating that the association between discrimination and smoking was stronger for male compared to female Caribbean Black youth. Although we did not find a significant interaction effect between gender and perceived discrimination for SU, our gender stratified analysis showed an effect of perceived discrimination on SU for males but not females. As effect sizes were small, and the results were not consistent for SU and smoking, the findings should be considered as suggestive. Multiple factors such as substance availability, acceptability, and peer norms influence risk of smoking and SU [75]. Although smoking and SU are correlated [76,77], smoking is legal with tobacco available in local markets; youth smoking is an early marker for later SU [78,79]. The distribution of smoking and SU also varies by gender.

Research conducted with samples from other ethnic groups has documented gender differences in the health correlates of perceived discrimination [18,19,43,46,49,80]. Most of this research has suggested that males might be more vulnerable to the association between discrimination and SU and psychopathology. This paper extends the existing research to Caribbean Black youth, one of the most understudied subgroups of Blacks in the U.S. Life experiences, history, and cultural values are different between Caribbean Black Americans and African Americans [81]. For instance, Caribbean Black Americans are employed more, and have higher income and SES than African Americans. They are more recent immigrants, with less recent history of slavery. They are also heavily concentrated in Florida, New York, and New Jersey. This pattern is very different from the southern states that have most African Americans. Immigrants from the Caribbean vary in their skill levels, racial composition, language background, as well as migration pathways to the United States, depending on origin country and period of arrival [82,83].

This study introduces discrimination as a potential risk factor for smoking in male Caribbean Black youth. This is particularly important because SU may be more consequential for Caribbean Blacks than African Americans [84]. For example, in a study among Caribbean Blacks but not African Americans, drug abuse disorder was a risk factor for suicidal thoughts. The study also showed an interaction between ethnicity and SU on suicide [84]. This is concerning given smoking and SU are not well-studied among Caribbean Blacks [85,86,87].

Males reported higher levels of perceived discrimination than female Caribbean Blacks. This pattern is in line with previous research showing higher report of discrimination by males than females across age groups and ethnicities [64,65]. In adult [15,46] and adolescent [18,60] African Americans, males report more perceived discrimination. The same pattern has been found for Arab American adults [43]. This can probably be explained by subordinate male hypothesis [28] which suggests males are the main target of discrimination by the dominant group. This is also supported by a recent study showing that White men hold more implicit bias against Black compared to White women [29].

Brodish et al. [45] have shown that perceived discrimination better predicts SU in male than female Blacks. Brondolo et al. [46] also showed that recent experience of discrimination is associated with an increased risk of smoking among male but not female Blacks. Perceived discrimination has also shown a stronger association with smoking in men than women [46]. Gender differences in the associations between environmental stressors such as discrimination and psychopathology are not limited to SU as they extend to psychological distress [43], depressive and anxiety symptoms [19,49,88], and major depressive disorders [80]. Although males seem to be vulnerable to depression in response to environmental stress [49], females seem to be more vulnerable to the effects of discrimination and environmental stress on obesity and eating disorders [59,89,90,91]. A study of Blacks following 14–21 year olds up until their 30s showed that after experiencing racial discrimination, Black men may have a higher tendency to turn to SU while Black women have a higher tendency to reduce exercise and physical activity [45]. This is also in line with Jackson’s theory that Black men turn to drugs and Black women turn to food to cope with stress, adversity, and perceived discrimination [92,93,94]. The results of the current study show some indication of this type of gender specific pattern of coping with discrimination.

As discrimination is a type of environmental stress, our findings are relevant to previous research that shows males may be more vulnerable to a wide range of environmental stressors than females on psychopathology and risk behaviors [19,49,80]. Similar to the pattern that we found here, environmental stress seems to be a stronger predictor of mental health outcomes for males than females across ethnic groups including but not limited to Blacks [18,19,43,46,49,80]. An increase in neighborhood stress predicted subsequent increase in depressive symptoms among male but not female Black youth [49]. Even outside the context of minority status, a 25-year longitudinal study of Black and White adults showed that environmental stress better predicts future risk of MDD in males than females [43]. Assari and Caldwell [80] used NSAL-Adolescents data and showed that higher level of neighborhood stress was associated with higher risk of MDD for African American males (OR = 1.41) but not African American females. The authors concluded that among African American youth, environmental stressors may have a more salient role for psychopathology of male than female Black youth [80]. There are other studies showing similar links between discrimination, environmental stress, and health outcomes in males and females [19]. For example, the association between perceived discrimination and suicidal ideation among Black youth was similar in males and females [19]. The current study and the prior research suggest that males may be more prone to various environmental stressors including but not limited to perceived discrimination resulting in psychopathology and health risk behaviors.

A wide range of social and cognitive factors may increase vulnerability of males compared to females to environmental stressors [18,43,45,49], that may result in an increased risk of SU in males compared to females. Masculine ideologies and gender norms may be the main mechanism by which males are more prone to perceived discrimination than females. Hegemonic masculinity ideologies (i.e., beliefs about men’s dominant position in the society and attention to hierarchy and power) restricts emotion expression and reduces service use when needed [47]. Research has also shown that masculine beliefs moderate the association between racial discrimination and depression among Black men [95]. A recent study showed that among Black males, high hegemonic masculine ideologies increase the magnitude of the association between neighborhood stress and depression among Black men [96]. Males, particularly those with higher hegemonic masculinities have higher restrictive emotionality, which may increase risk of depression [47]. Higher self-reliance that follows masculine ideologies increases risk of depression and lowers chance of help seeking when needed [95]. Gender norms and ethnic identity may alter the vulnerability of Blacks to discrimination and other types of environmental stress [25].

Our results should be interpreted alongside research about risk associated with other sociodemographic factors on Blacks’ vulnerability to discrimination. For example, high SES measured by educational attainment [15] or income [97] have been shown to increase Blacks’ vulnerability to perceived discrimination. Using data from NSAL-A, high compared to low SES was associated with more vulnerability to perceived discrimination on depression in Black youth [97]. Hudson et al. [98], have also shown that SES may alter the association between perceived discrimination and depression, with discrimination being more closely linked to poor mental health at higher levels of SES. Hudson et al. [99], have also shown that high SES predicts higher levels of perceived discrimination among African Americans. These studies and our results collectively suggest that consequences of discrimination depend on the intersection of race, ethnicity, gender, and class.

Gender differences in coping with stress [25,100] may explain the gender differences in the association between perceived discrimination and poor mental health. Women may have a higher tendency to use avoidant coping (characterized by avoidance behavior) compared to men [100]. In contrast, men may have a higher tendency to use confrontational or effortful coping including but not limited to using John Henryism (i.e., a strong behavioral predisposition towards active and effortful coping with environmental stressors such as discrimination) [101,102] resulting in worse health consequences for Black males than females [101]. Women also have a higher tendency to turn to religion and social support to cope with environmental stressors such as discriminations [103,104]. Overall, compared to men, women more frequently seek social support and care from various sources including family, friends, and religion when needed. Such gender differences in coping have important implications for the link between stress and health for women [105,106,107,108].

Gender differences exist in sources of discrimination in Blacks, which may also be present for Caribbean Blacks, and may explain our findings. Males and females may experience different types of discrimination (e.g., invalidation/devaluation vs. threat-based, i.e., discrimination due to fear). In addition to experiencing considerable threat-based discrimination, survival becomes a major priority for Black males who live in violent neighborhoods [35,49]. Black males are more commonly discriminated against by U.S. institutions such as education systems, labor markets, as well as correctional systems [34,65,109]. Police brutality, mass incarceration, stop and frisk, and school-to-prison pipeline disproportionately affect and influence Black males [110,111,112]. A recent study showed that discrimination fully mediates the association between incarceration history on poor mental health of African American males, emphasizing unique experiences of Black males regarding discrimination that may be qualitatively different from that of females [34].

An unexpected finding of the current study was the higher levels of SU and smoking in female than male Caribbean Black youth. This is in contrast to most of the literature showing that SU is more common in males than females [113,114,115]. There are, however, relevant studies showing high vulnerability of Caribbean Black females compared to Caribbean Black males. For instance, high education is associated with more (not less) suicidality in Caribbean Black females [84]. Caribbean Black females not males show additional vulnerability to the association between perceived discrimination and depression if they see race as a barrier against upward social mobility [116]. As females were significantly older than males in the current study, and age emerged as a significant predictor of SU and smoking, one likely explanation for the higher rates of SU among girls relative to boys is age. Gender, however, stayed as a significant predictor of SU in regression models that controlled for age. Future research is needed on higher risk of SU, smoking, and other vulnerabilities for Caribbean Black females.

## 5. Limitations

Our study is subject to a few limitations. First, we used a cross-sectional design, so our findings suggest associations not causations. Second, although the data are from 2003, we are not aware of any other national study of Caribbean Blacks that has measured discrimination and substance use. The NSAL remains a rare mental and behavioral survey of ethnically diverse sample of Blacks in the U.S. We do not see any reasons that suggest the results would be less relevant to the life of Blacks today than 15 years ago. The topic of discrimination, the health consequences of such exposure, and gender differences in vulnerability are still very relevant to the daily life of Blacks in the U.S. The existing knowledge gap on such important topic justified our use of 15 years old data. Although discrimination and prejudice is declining over long period of time [117], recent political changes in U.S. has increased exposure to discrimination and racism [118,119]. Third, our study is subject to measurement bias. We used a single item to measure lifetime smoking, which does not distinguish between experimental and chronic smoking. Single item measures are commonly used to assess smoking in large-scale national surveys, and have good validity and reliability when measuring lifetime use in youth. Lifetime smoking during adolescence is a risk factor for adult SU [120]. Another measurement concern is that males and females may differ in reporting discrimination and SU [28,121,122]. In addition, our smoking measure did not differentiate type of tobacco used. Risk factors for use of different tobacco types differ and may not have homogeneous associations with perceived discrimination. This study exclusively focused on the aggregate measure of SU and smoking but not on alcohol or specific illicit substances such as marijuana, or opiates. Due to the low frequency of each substance, we were not able to model each substance individually. Instead, we calculated a composite measure for illicit SU regardless of its type. Fourth, we may have residual confounding, as we also did not measure variables such as depression and parental SU. Future research should test the role of nativity and years since immigration on the association between discrimination and SU. Fifth, the sample size was not balanced across gender groups. However, lack of an association in females may not be due to statistical power. In fact, SU was more common among female than male Caribbean Black youth, which needs more research. Thus, the results should be replicated in other settings and samples, particularly using current use, and more comprehensive measures. Despite these limitations, the current findings extend the literature. We believe this was one of the first studies to explore gender differences in the associations between perceived discrimination and SU in a national sample of Caribbean Black youth. Most previous research on this topic has been conducted among African Americans [45,46].

## 6. Implications

Our results have implications for policy and practice related to mental health of Caribbean Black youth in the United States. It is not easy to reduce discrimination against minorities. Many anti-discriminatory laws are already in place, but discrimination remains rampant [16,17]. The New Deal and civil right laws [123] have reduced overt discrimination, however, still there is a need to enhance racial/ethnic equity in the United States. Instead of designing and implementing new legislations, the emphasis should be placed on enforcement of anti-discriminatory laws that already exist. Such policies have the potential to reduce discrimination at all levels and institutions against all groups including Caribbean Black youth. Multilevel interventions should reduce discrimination at schools, labor market, and criminal justice system. Meanwhile, interventions and programs should help Blacks, particularly males, to enhance their coping skills to better respond to discrimination and environmental stressors in their lives.

Promotion of family engagement for men provides a unique opportunity to promote mental and behavioral health of Black males [47,124]. Mental health practitioners and health care providers should consider discrimination and trauma as salient determinants of behavioral health problems for Caribbean Black males. As a result, evaluation of exposure to environmental and interpersonal stressors should become an essential part of SU prevention programs that reach Black males, especially Caribbean Black makes. Further, a thorough evaluation of stressors should be a part of typical well-child care for Caribbean Black adolescents in the U.S.

## 7. Future Research

Despite significant *p* values [125,126], the magnitude of the associations reported here are still small. More research is needed on relative importance of discrimination on SU in subgroups of Blacks. Future research is also needed to understand why discrimination is more strongly associated with SU and other behavioral health problems in males than females. Research may investigate the role of effective coping, health behaviors, social support, and help seeking behaviors to explain the observed gender differences. Gender norms, social norms, ethnic identity, and attribution may also play a role in explaining gender differences in the health correlates of perceived discrimination. It is unknown whether nativity and years since immigration to U.S. modify the links between discrimination on SU. Finally, as race and SES overlap, future research should test whether discrimination interacts with SES in this population [97].

## 8. Conclusions

Our findings are suggestive of potential gender differences in the association between perceived discrimination and SU among Caribbean Black youth. Although the results are preliminary, Caribbean Black males may be more vulnerable to the effect of perceived discrimination on smoking (and to some extent SU), compared to Caribbean Black females. Policies and practices that aim to reduce harms of SU and smoking in subgroups of Blacks. The role and experience of discrimination for male Caribbean Black youth is different. Substance use prevention programs for Caribbean Black boys may benefit from addressing discrimination.

## Figures and Tables

**Table 1:** Descriptive statistics in the pooled sample of Caribbean Black youth and based on gender

	All		Males		Females	
	(*n* = 360)		(*n* = 165)		(*n* = 195)	
	Mean	95% CI	Mean	95% CI	Mean	95% CI
**Age** *	15.21	15.08–15.34	14.80	14.59–15.01	15.55	15.44–15.65
**Poverty Index**	4.19	3.61–4.77	4.43	3.58–5.27	4.00	3.62–4.39
**Discrimination** *	5.22	4.03–6.41	6.13	4.25–8.01	4.48	3.75–5.22
	**%**	**95% CI**	**%**	**95% CI**	**%**	**95% CI**
**Smoking** *						
No	69.73	65.72–73.45	73.05	47.17–89.16	67.02	49.86–80.59
Yes	30.28	26.55–34.28	26.95	10.84–52.83	32.98	19.41–50.14
**Substance Use** *						
No	79.45	66.77–88.15	83.66	57.80–95.03	76.03	70.16–81.05
Yes	20.55	11.85–33.23	16.34	4.97–42.20	23.97	18.95–29.84

* *p* < 0.05 for comparison of males and females. CI: Confidence Interval.

**Table 2:** Correlations matrix between the study variables among Caribbean Black youth overall and by gender

	1	2	3	4	5
	Age	Poverty	PD	Smoking	SU
**Total Sample**					
1 Age	1.00				
2 Poverty	0.03	1.00			
3 Perceived Discrimination (PD)	0.13	0.14	1.00		
4 Smoking	0.20 *	0.04	0.11	1.00	
5 Substance Use (SU)	0.21 *	−0.05	0.15	0.40 *	1.00
**Females**					
1 Age	1.00				
2 Poverty	−0.03	1.00			
3 Perceived Discrimination (PD)	0.10	0.21 *	1.00		
4 Smoking	0.15	0.03	0.14	1.00	
5 Substance Use (SU)	0.23 *	0.01	0.10	0.39 *	1.00
**Males**					
1 Age	1.00				
2 Poverty	0.10	1.00			
3 Perceived Discrimination (PD)	0.18	0.05	1.00		
4 Smoking	0.25 *	0.05	0.08	1.00	
5 Substance Use (SU)	0.20 *	−0.11	0.18	0.42 *	1.00

* *p* < 0.05.

**Table 3:** Logistic regressions on the association between perceived discrimination and substance use among Caribbean Black youth

	Model 1		Model 2		Model 3		Model 4	
	Main Effects		Main Effects + Interaction		Males		Females	
	OR	95% CI	OR	95% CI	OR	95% CI	OR	95% CI
Gender (Male)	0.68	0.32–1.44	0.11 *	0.01–1.13	-	-	-	-
Age	1.55 *	1.04–2.30	1.60 #	0.98–2.61	1.03	0.57–1.85	2.17	0.58–8.13
Poverty	1.00	0.39–2.61	1.03	0.39–2.73	1.19	0.76–1.85	0.95	0.32–2.79
Discrimination	1.15 *	1.02–1.29	1.02	0.92–1.14	1.32*	1.05–1.67	1.02	0.91–1.15
Discrimination × Gender	-	-	1.32 #	0.94–1.86	-	-	-	-

Outcome: substance use # *p* < 0.1; * *p* < 0.05.

**Table 4:** Logistic regressions on the association between perceived discrimination and smoking among Caribbean Black youth

	Model 1		Model 2		Model 3		Model 4	
	Main Effects		Main Effects + Interaction		Males		Females	
	OR	95% CI	OR	95% CI	OR	95% CI	OR	95% CI
Gender (Male)	0.81	0.27–2.39	0.25 *	0.08–0.78	-	-	-	-
Age	1.31 *	1.04–1.64	1.34 *	1.07–1.69	1.17	0.47–2.91	1.46 #	0.98–2.18
Poverty	1.09	0.89–1.32	1.10	0.91–1.35	1.30	0.90–1.86	1.03	0.76–1.40
Perceived Discrimination	1.07	0.77–1.49	0.97	0.70–1.34	1.18	0.88–1.56	0.97	0.72–1.30
Perceived Discrimination × Gender	-	-	1.23 *	1.07–1.41	-	-	-	-

Outcome: Smoking. # *p* < 0.1; * *p* < 0.05.
